# The updated global burden of chronic kidney disease: one death every 20 seconds

**DOI:** 10.1093/ndt/gfag040

**Published:** 2026-02-25

**Authors:** Alberto Ortiz, Jennifer S Lees, Roser Torra, Vianda S Stel, Anneke Kramer, Patrick B Mark

**Affiliations:** Division of Nephrology and Hypertension, IIS-Fundación Jiménez Díaz-Universidad Autónoma Madrid, Spain; Department of Medicine, Universidad Autonoma de Madrid, Madrid, Spain; School of Cardiovascular and Metabolic Health, University of Glasgow, Glasgow, UK; Glasgow Renal and Transplant Unit, Queen Elizabeth University Hospital, Glasgow, UK; Nephrology Department, Fundació Puigvert, Institut de Recerca Sant Pau (IR-Sant Pau), Departament de Medicina, Universitat Autònoma de Barcelona, Barcelona, Spain; ERA Registry, Department of Medical Informatics, Amsterdam UMC – Location University of Amsterdam, Amsterdam, The Netherlands; Amsterdam Public Health Research Institute, Quality of Care, Amsterdam, The Netherlands; ERA Registry, Department of Medical Informatics, Amsterdam UMC – Location University of Amsterdam, Amsterdam, The Netherlands; Amsterdam Public Health Research Institute, Quality of Care, Amsterdam, The Netherlands; School of Cardiovascular and Metabolic Health, University of Glasgow, Glasgow, UK; Glasgow Renal and Transplant Unit, Queen Elizabeth University Hospital, Glasgow, UK

**Keywords:** chronic kidney disease, disease burden, epidemiology, kidney replacement therapy, mortality

## Abstract

In 2024, Global Burden of Disease (GBD) updated the forecast of global cause-specific age-standardized death rates. Chronic kidney disease (CKD) was forecast to increase by over 30% from 2022, while stroke and ischaemic heart disease would decrease by more than 40%. In western Europe, CKD would become the third cause of death by 2050. While proactive primary prevention has been key to addressing the burden of stroke and ischaemic heart disease, its role in CKD has been often neglected. In 2025, the World Health Organization and Kidney Disease: Improving Global Outcomes produced documents emphasizing maintenance of kidney health and GBD updated the global epidemiology estimates for CKD and kidney replacement therapy (KRT). The new data support the previous estimate that there are 850 million people with kidney disease globally, of whom 4.6 million are on KRT, and 1.5 million people annually (one every 20 seconds) die from CKD. In Europe, an estimated 93.1 million adults have CKD, of whom 750 000 are on KRT, and 210 000 people (one every 2.5 minutes) die from CKD. Of relevance for public health planning, diabetes and hypertension accounted for ≤30% of prevalent KRT in Europe. GBD estimates extend data currently available through the European Renal Association Registry by including additional countries where prevalence of KRT is high: Germany, for example, is home to over 77 000 people on KRT. Overall, these updated data confirm the severity of the global and European CKD burden and call for urgent action to develop novel strategies that expand beyond screening, early diagnosis and treatment of CKD to also encompass proactive primary prevention.

## CONFIRMATION OF THE STRIKING NUMBER OF 850 MILLION PEOPLE WITH KIDNEY DISEASE

Chronic kidney disease (CKD) is diagnosed when there is abnormality of kidney structure or function persisting for longer than 3 months, with deleterious implications for health [1]. Among other criteria, CKD can be diagnosed when the glomerular filtration rate (GFR) falls below 60 mL/min/1.73 m^2^ or the urinary albumin–creatinine ratio is ≥30 mg/g. This latter point is commonly forgotten or ignored, with concerningly low rates of albuminuria testing even among those most likely to have a positive test (e.g. patients with diabetes). Such a high-risk person who has not undergone assessment of albuminuria may already have a 100-fold higher risk of kidney failure requiring kidney replacement therapy (KRT) [2, 3] while remaining undiagnosed and untreated despite the availability of interventions that may delay kidney failure by several decades [4].

The burden of CKD has long been overlooked. As a silent disease, awareness in the general population is suboptimal. The assumption in a worst-case scenario (progression to kidney failure) that KRT in some manner is a satisfactory replacement for kidney function may have brought complacency to policy makers and the healthcare establishment. However, KRT by either dialysis or transplantation does not restore all kidney functions. As an example, the anti-ageing function of the kidneys, dependent in part on the kidney production of molecules such as Klotho or betaine, is not fully restored [5, 6]. Thus, accelerated biological aging with premature death is a key feature of CKD [7] that continues to progress in people on KRT. As a consequence, in young men and women on dialysis, remaining life expectancy may be over 40 years shorter than in the general population [8] and even for young individuals with a kidney transplant, their life expectancy is approximately 20 years shorter than that of someone in the general population.

To help create awareness about this unjustifiably overlooked health issue [9], in 2019, the European Renal Association (ERA), International Society of Nephrology (ISN) and American Society of Nephrology (ASN) agreed uniform estimates of the kidney disease burden for advocacy and clarity of communication: 850 million worldwide people have kidney diseases, of whom 843 million were estimated to have CKD and 7 million other forms of kidney disease (acute kidney injury, acute kidney disease) [10]. The latest iteration of the Global Burden of Disease (GBD) study used 2023 global data and was published in November 2025. Overall, findings are aligned with ERA-ISN-ASN estimates: worldwide, the number of adults with CKD more than doubled from 1990 to reach 788 million (95% uncertainty interval 743–843 million) in 2023 [11]. In Europe, 93.1 (87.5–99.0) million people were estimated to have CKD, 47% of them in Western Europe. The European countries with the highest numbers of people with CKD were Russia, Türkiye, Germany, the UK and Ukraine (Fig. 1). For these estimates, we used GBD data for Western, Central and Eastern Europe and Türkiye, as all these countries have European territory.

**Figure 1:**
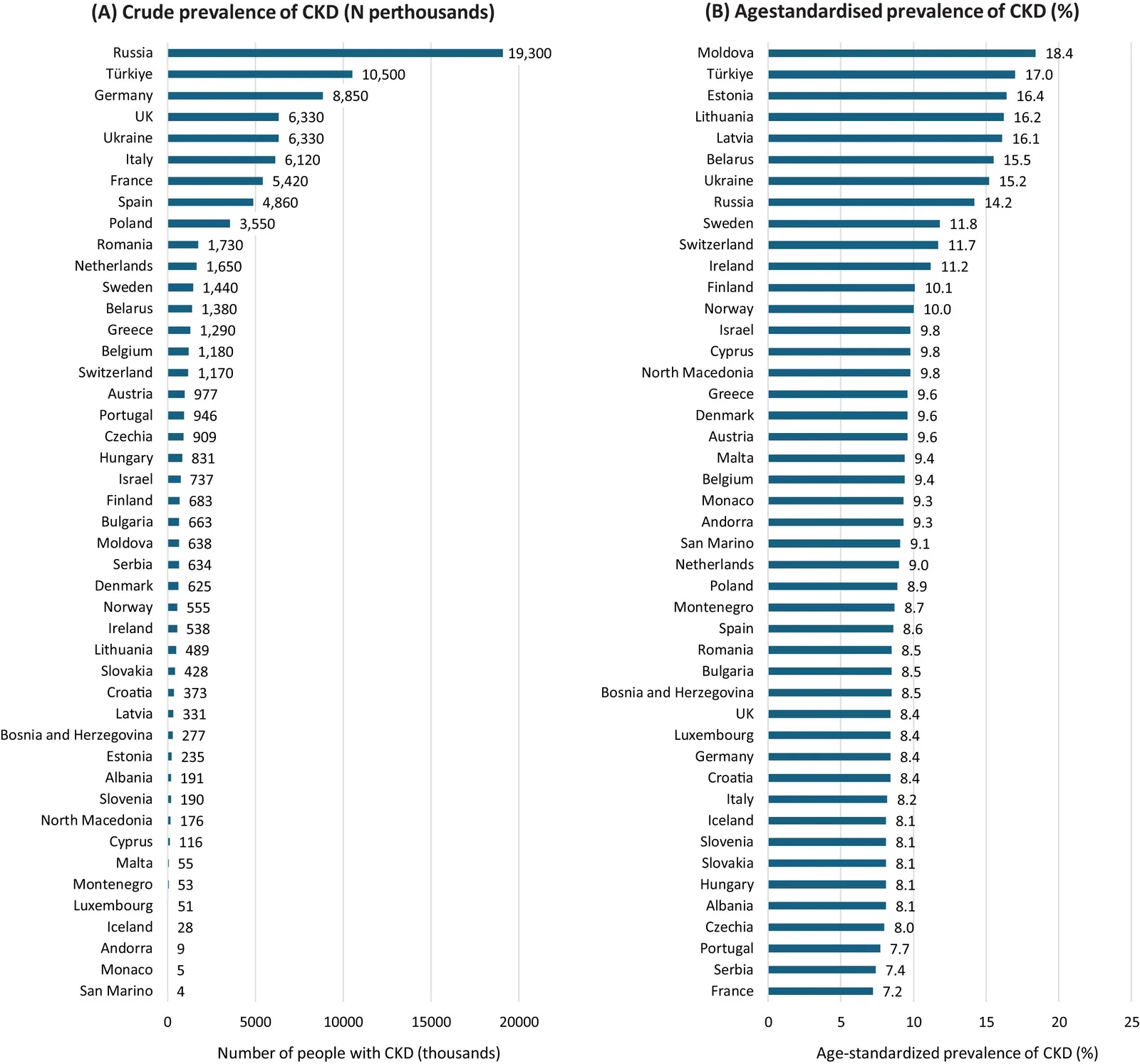
Estimated prevalence of CKD in adults >20 years old by country in Europe according to GBD 2023. (**A**) Crude prevalence as estimated number of adults >20 years old with CKD by (**B**) age standardized prevalence of CKD in adults >20 years old by country in Europe according to GBD 2023. A country was considered European if at least part of its territory was located in Europe (e.g. Russia and Türkiye).

The number of people with CKD in a given country will largely depend on the size of the population. Crude prevalences allow comparisons between countries with different population sizes. The global crude prevalence of CKD in adults was 14.6% (13.8% to 15.6%), a 18.9% (16.7% to 20.9%) increase from 1990. In Western Europe, the crude prevalence of CKD was 12.4% (11.7% to 13.2%) (a 19.5% increase from 1990), in Central Europe 11.1% (10.4% to 11.8%) (a 25.9% increase from 1990), in Eastern Europe 17.2 (16.2% to 18.4%) (a 14.6% increase from 1990) and in Türkiye 17.2% (16.0% to 18.5%) (a 19.4% increase from 1990). The European countries with the highest crude prevalences were Estonia, Latvia and Moldova.

For an age-associated condition, as is CKD, crude prevalence may be influenced by the age pyramid of the population: they may be higher in countries with age pyramids skewed towards older age. Age-standardized prevalences allow comparison of countries with different age pyramids. The global age-standardized prevalence of CKD in adults was 14.2% (13.4% to 15.2%). In Western and Central Europe it was 8.5%, in Eastern Europe 14.6% and in Türkiye 17.0%, all essentially unchanged since 1990, reflecting that to some extent the increase in crude CKD prevalence reflects the population ageing. This is in keeping with other analyses suggesting that age standardized CKD prevalence is projected to remain unchanged up until 2050 [12] and previous reports demonstrating that whilst the crude global prevalence of CKD increased by one-third between 1990 and 2016, the age-standardized prevalence remained relatively stable (increasing from 4.04% to 4.06%) [13]. The European countries with the highest age-standardized prevalences in adults were Moldova, Türkiye and Estonia (Fig. 1).

## ONE DEATH FROM CKD EVERY 20 SECONDS

In 2023, CKD was the ninth leading cause of death globally, accounting for 1.48 million (1.30–1.65) deaths, i.e. one death every 20 seconds. It was also the 12th leading cause of disability-adjusted life years (DALYs), with an age-standardized DALY rate of 769.2 (691.8–857.4) per 100 000. In Europe, 210 000 people died from CKD in 2023, i.e. one death every 2.5 minutes. The highest number of deaths was observed in Germany (42 398 deaths estimated to be due to CKD), Italy (26 825 deaths estimated to be due to CKD) and Türkiye (19 325 deaths estimated to be due to CKD) (Fig. 2A). There were more deaths from CKD in women than in men. For example, in Germany, there were around 25 000 women deaths estimated to be due to CKD. The highest age-standardized death rates were found in Türkiye, Estonia, Cyprus and Greece (Fig. 2B). Notably, several Eastern European countries with high age-standardized prevalence of CKD (e.g. Moldova, Belarus, Ukraine, Russia) had relatively low high age-standardized death rates estimated to be due to CKD, likely because of high premature mortality rates due to other causes. Trends in the mortality rates attributable to CKD in Europe are shown in Fig. 3 [14]. The age-standardized DALY rates were 352.7 (304.4–394.0) per 100 000 for Western Europe, 380.3 (344.6–418.5) per 100 000 for Central Europe, 281.0 (243.8–318.0) per 100 000 for Eastern Europe and 979.3 (805.7–120.6) per 100 000 for Türkiye.

**Figure 2:**
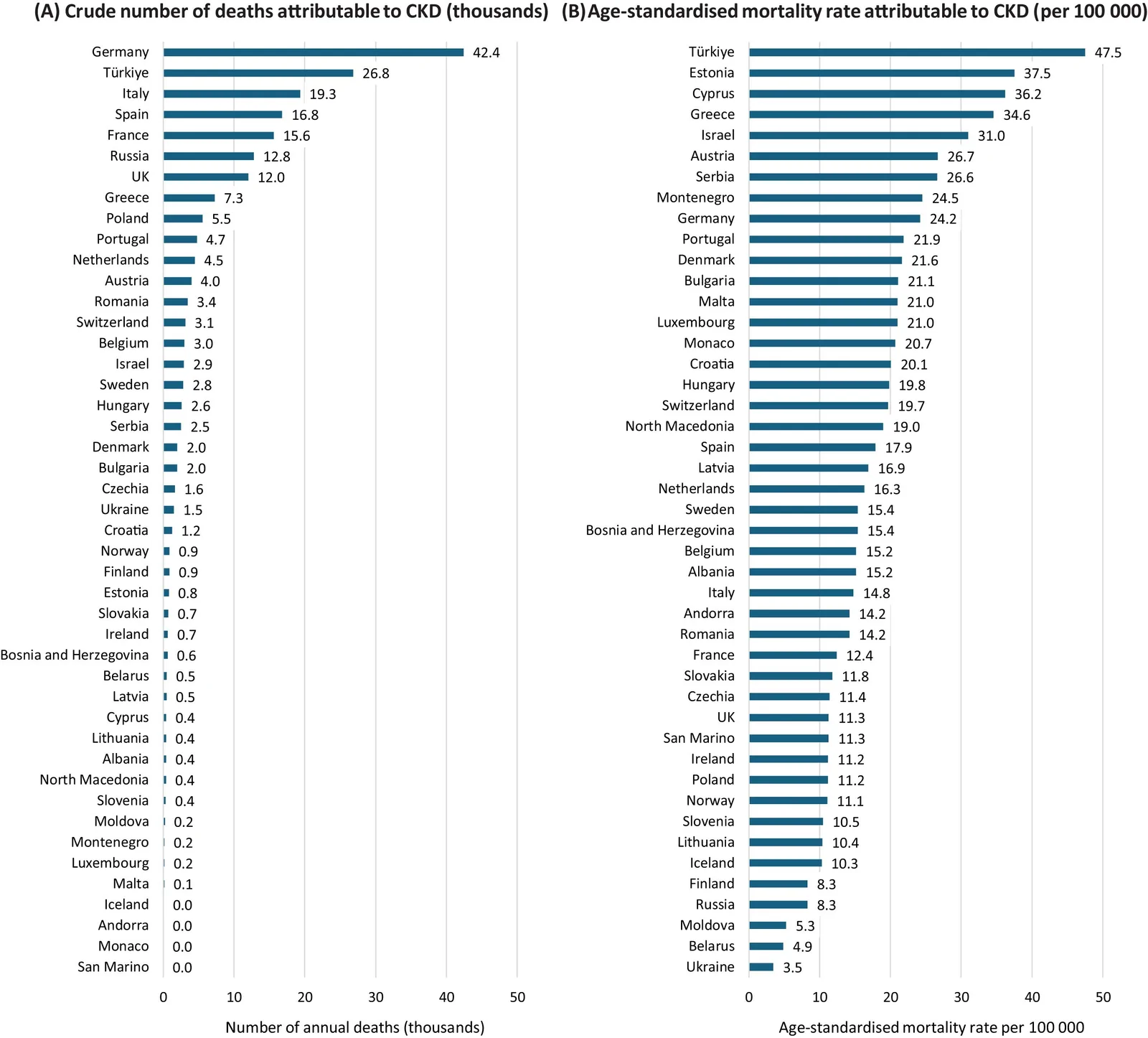
Estimated number of deaths in 2023 attributable to CKD in all ages in European countries. (**A**) Annual number of deaths (thousands). (**B**) Age-standardized rate per 100 000.

**Figure 3:**
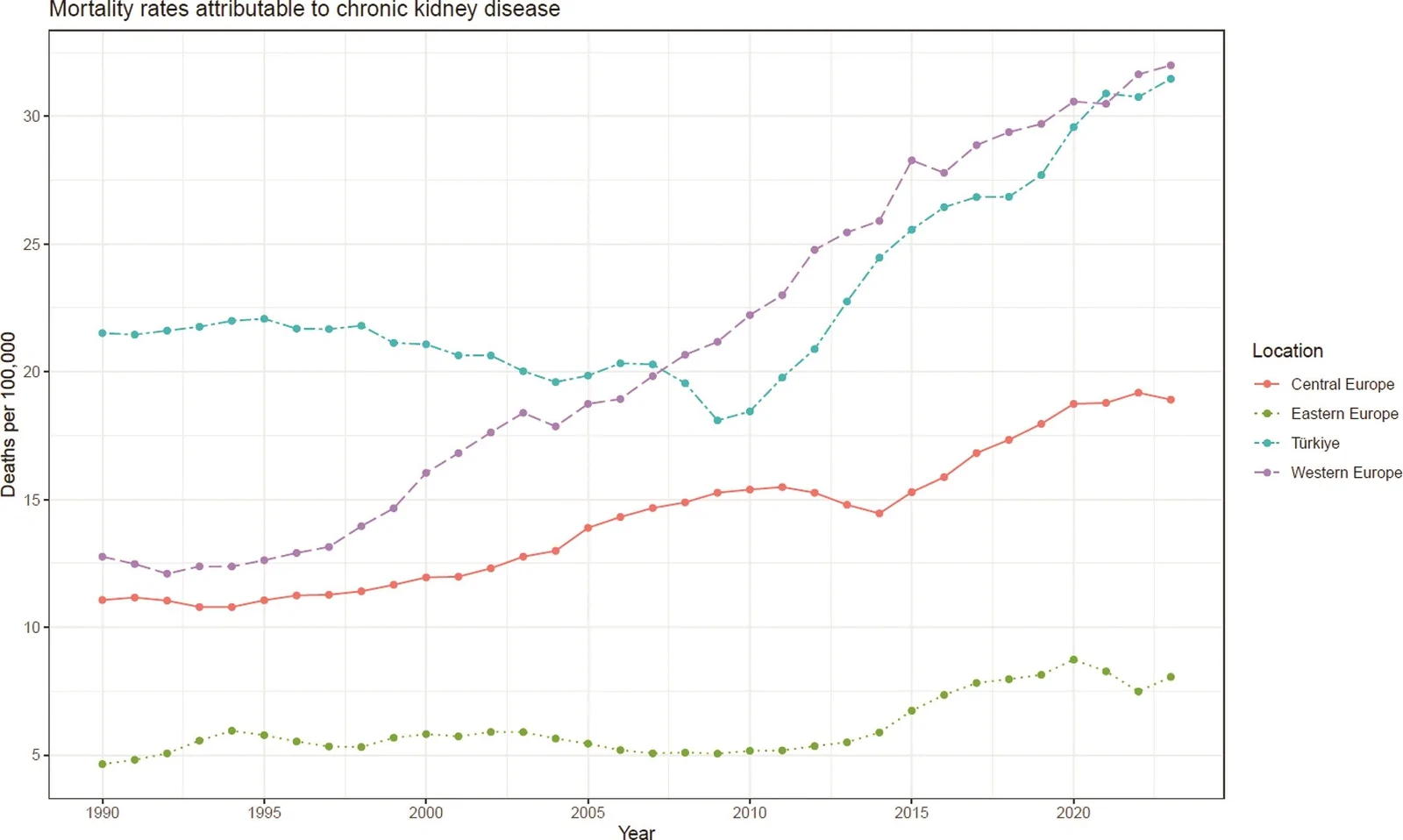
Trends in estimated mortality attributable to CKD 1990–2023 in European countries. Figure generated from GBD Compare for Western Europe, Central Europe, Eastern Europe and Türkiye [14]. GBD Compare Data Visualization (https://vizhub.healthdata.org/gbd-compare, accessed 11/12/25).

While the burden of CKD was large in 2023, the forecast for 2050 is even more concerning [15]. GBD forecasts that CKD will become the 3rd leading cause of death in Western Europe by 2050 [a 35.9% (95% uncertainty intervals, UI, 16.3% to 59.7%) increase in age-standardized death rate from 2022], the 11th leading cause of death in Central Europe [35.3% (–8.93% to 96.2%) increase from 2022], the 18th leading cause of death in Eastern Europe [40.2% (–1.02% to 95.9%) increase from 2022] and the 4th leading cause of death in Türkiye [3.9% (–27.4% to 58.7%) increase from 2022]. As a comparator, the age-standardized death rate for ischaemic heart disease is forecast to decrease by 48% to 58%, stroke by 49% to 54% and diabetes by 27% to 35% in these regions during the same period. This would make CKD the most adverse contributor to the cardiovascular–kidney–metabolic (CKM) syndrome, unless prevention and early treatment approaches are implemented similar those that already used for other CKM conditions.

The projected increase in age-standardized death rates from CKD may appear to be at odds with the stable global age-standardized prevalence of CKD in the past three decades. A potential driver of this discrepancy is the projected decreasing mortality from other causes in which a combination of primary prevention (e.g. cardiovascular disease) and early diagnosis and treatment (universal screening for certain cancers or the existence of treatable pre-conditions) is practiced, unlike for CKD.

## UPDATED KRT EPIDEMIOLOGY

Also in 2025, GBD reported 2023 global epidemiology data for KRT [16]. Globally, 4.6 (95% UI 4.2–5.1) million people were on KRT, a near 3-fold increase from 1.6 (95% UI 1.4–1.8) million in 1990. The age-standardized prevalence of KRT was 50.7 (46.1–56.0) per 100 000 population. This corresponds to crude and age-standardized prevalence of dialysis of 44.3 (39.8–50.1) and 39.3 (35.6–44.4), respectively. Corresponding figures for kidney transplantation were 12.7 (11.2–14.6) and 11.3 (10.1–13.1). Of 15 countries with a kidney transplant prevalence higher than the dialysis prevalence, 11 were in Europe [16]. Type 2 diabetes and hypertension were among the leading causes of kidney failure requiring KRT globally.

The highest prevalence of KRT was found in high-income regions, since access to KRT, particularly kidney transplantation, is limited in many parts of the world, for example notably in sub-Saharan Africa and South Asia. Indeed, there were discrepancies between KRT and CKD prevalence across the world and within Europe (Fig. 4).

**Figure 4:**
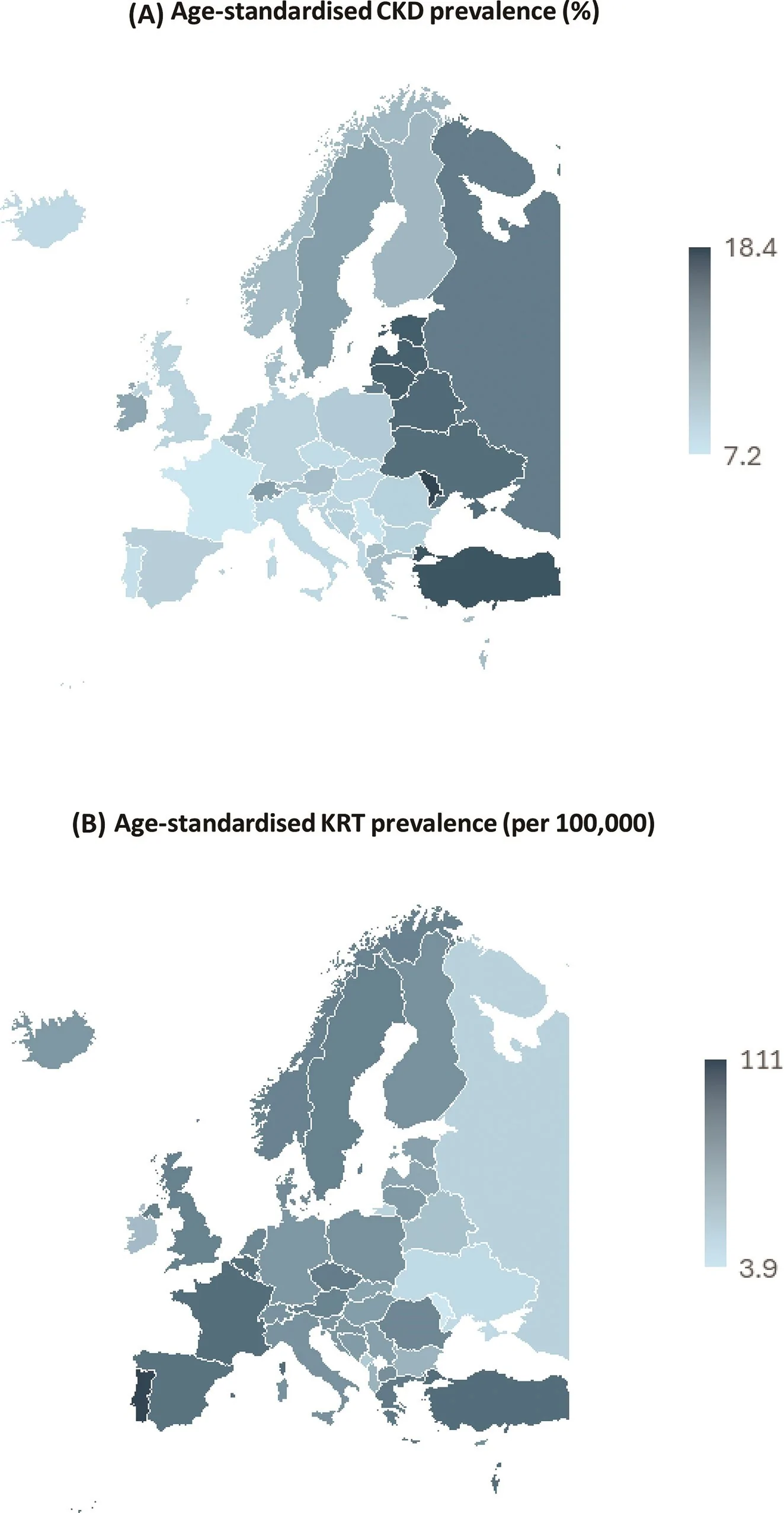
Geographical distribution of age-standardized CKD and KRT prevalence according to GBD 2023. (**A**) Geographical distribution of age-standardized CKD prevalence. (**B**) Geographical distribution of age-standardized KRT prevalence.

The ERA Registry reports annually on the incidence and prevalence of both components of KRT—dialysis and transplantation—in contributing countries and regions [17, 18]. However, not all countries are included in this report. The largest of the non-contributing countries is Germany [19]. While the GBD report on the epidemiology of KRT relied heavily on ERA Registry data, it also covered the blank spaces in Europe, including Germany, which represents one of the European countries with the most people on KRT (Fig. 5). Overall, the three European countries with the highest age-standardized prevalence of KRT were Portugal, Türkiye and France, followed closely by Belgium and Spain. The number of prevalent patients on KRT was highest in France, Türkiye and Germany followed closely by Italy and Spain.

**Figure 5:**
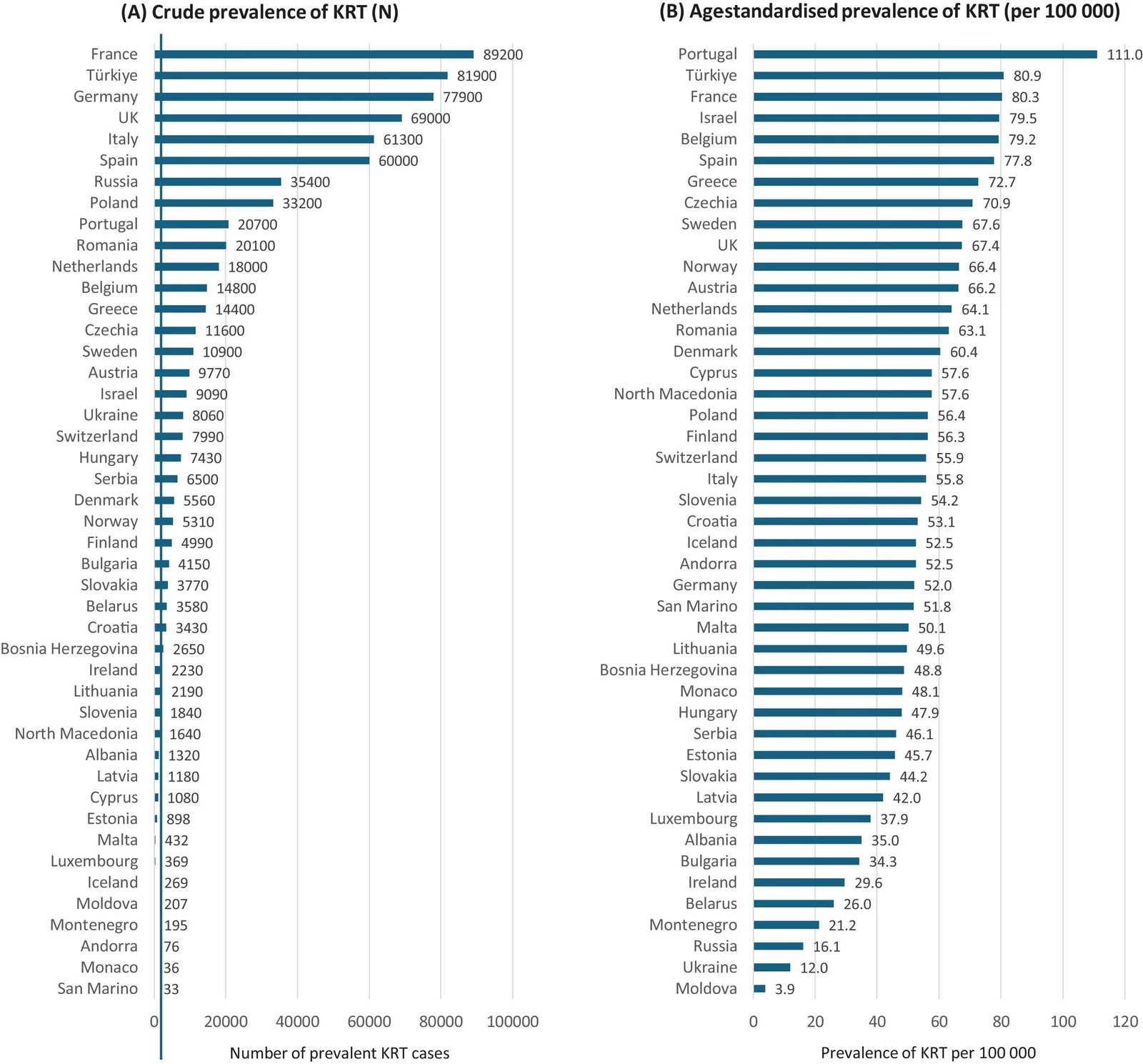
Prevalence of kidney replacement therapy in all ages in European countries, according to GBD 2023. (**A**) Number of prevalent cases. (**B**) Age-standardized prevalence per 100 000.

As previously reported by the ERA Registry [20, 21], there were large differences in the prevalence of KRT in different European countries, a 28-fold difference between Portugal (age-standardized prevalence 111 per 100 000) and Moldova (age-standardized prevalence 3.9 per 100 000) (Fig. 5). While socioeconomic factors limiting access to KRT may play a role, other factors may contribute, as large differences are also observed within countries [22]. Additionally, there was a 61-fold difference in the kidney transplant prevalence/dialysis prevalence ratio between Norway (2.44) and Slovakia (0.04) (Fig. 6). Understanding potential contributors to regional differences may identify best practices that improve patient outcomes. In this regard, these numbers serve to benchmark healthcare systems regarding kidney failure outcomes.

**Figure 6:**
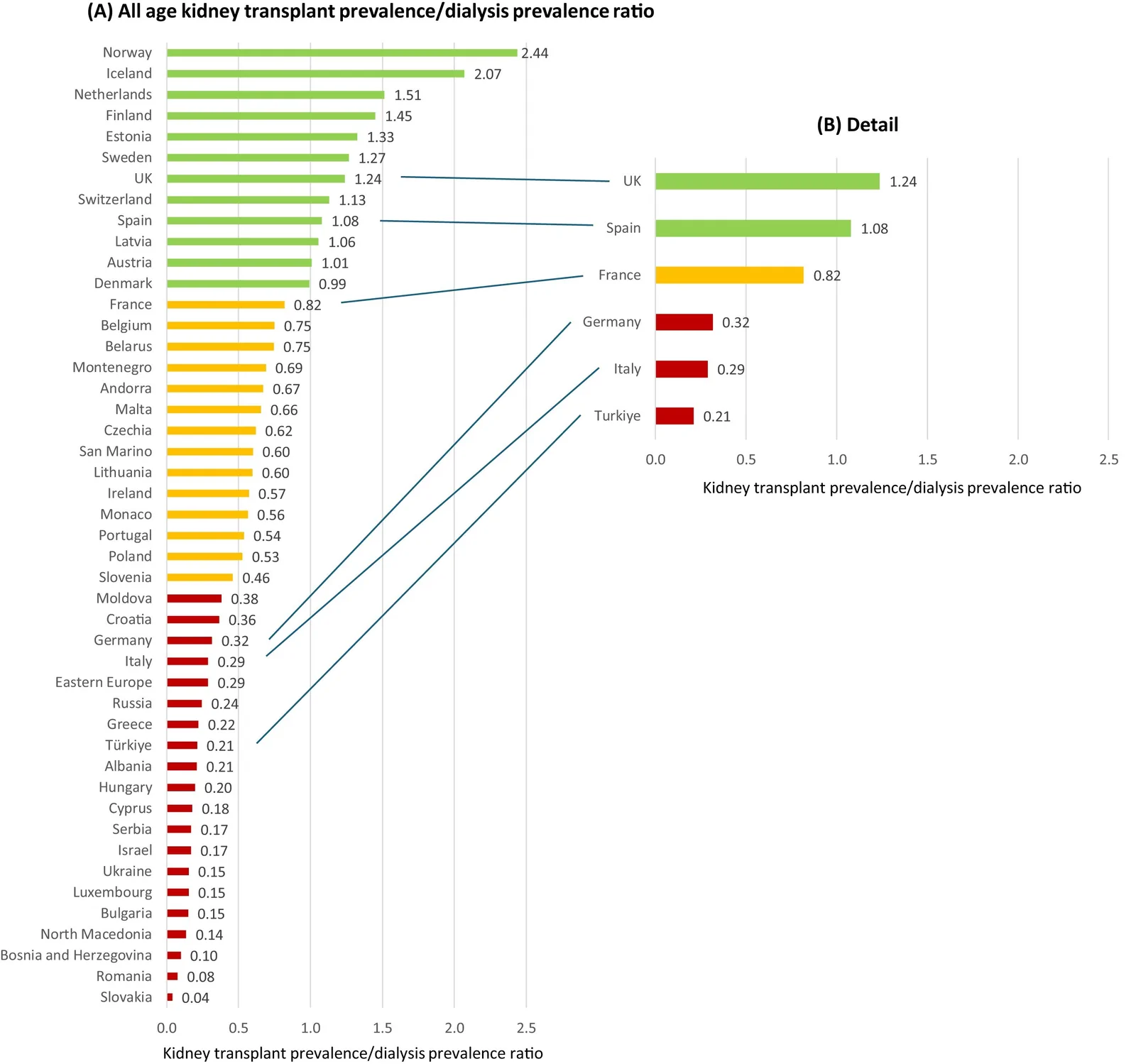
Ratio of kidney transplant prevalence to dialysis prevalence in all ages In Europe according to 2023. (**A**) Ratio of kidney transplant prevalence to dialysis prevalence. (**B**) Detail: European countries with highest KRT prevalence in 2023.

The contemporary, all age prevalence data available in Germany for both dialysis [70.4 (58.2–88.7) per 100 000] and kidney transplantation [22.2 (20.2–24.3) per 100 000] complement prior estimates and allow comparisons with other large European countries [23]. While the prevalence of dialysis is in the same range as for other large European countries (range 61 to 81 per 100 000 for Türkiye, Italy, France and Spain), the prevalence of kidney transplantation is around 3-fold lower than in France and Spain. This results in a kidney transplantation/dialysis prevalence ratio of 0.32, as compared with 0.82 to 1.24 for France, Spain and the UK (Fig. 5). It should be a wake-up call for German nephrologists and their healthcare system, while another high-income country, Italy, shares a similar problem—this was already hinted by the Italian regional data contributed to the ERA Registry. A call should be made for greater transparency regarding the access to kidney transplantation across countries that are currently not sharing these data, in accordance with the WHO Declaration on kidney health 2025 [24].

The change in KRT prevalence over 33 years (1990–2023) was heterogenous between countries and for dialysis and kidney transplantation [16]. In all countries, all age KRT prevalence increased faster than age-adjusted prevalence, implying that an increased prevalence of KRT in older people was the main driver of KRT growth. In most countries dialysis prevalence grew faster than kidney transplantation prevalence. In 14 countries, age-adjusted kidney transplantation prevalence grew faster than dialysis prevalence, and in 7 countries that was also the case for all age prevalence change. The largest increase in kidney transplantation prevalence as compared with dialysis prevalence was observed in the Netherlands, Slovenia and Iceland. Portugal, Romania, Greece and Türkiye experienced the largest growth in all age dialysis prevalence (>65 per 100 000), while Türkiye, Romania, Portugal and Israel grew the most in age-adjusted terms (≥35 per 100 000). Portugal and Spain experimented the largest growth in kidney transplant prevalence, both in all age (>35 per 100 000) and age-adjusted terms (19 to 21 per 100 000).

The geographical distribution of age-standardized CKD prevalence was almost specular to the geographical distribution of age-standardized KRT prevalence, potentially pointing to unmet healthcare needs (Fig. 4) [11, 16]. Thus, age-standardized CKD prevalence was highest in Easten Europe while age-standardized KRT prevalence was highest in Central and Western Europe.

A relevant insight for public health planning refers to the cause of kidney failure leading to KRT. Diabetes and hypertension were only minor contributors to the burden of KRT in Europe, accounting for <30% of prevalent cases, as compared with 69% in the USA and 48% in the world [16]. In line with ERA Registry findings, glomerulonephritis and ‘other’ were more relevant causes of KRT in Europe, and the ERA Registry had identified inherited kidney disease (IKD)/congenital anomalies of the kidney and urinary tract (CAKUT) as the largest contributor to ‘other’ in prevalent patients [25].

Several factors may influence KRT epidemiology, including expanded access to KRT, especially for the elderly and multimorbid, as well as evolving practice related to the timing of KRT initiation [26]. The latter is difficult to assess due to evolving nature of methods for GFR assessment. However, it may have increased during the 1990s and then decreased again during the 2000s. As examples, in the UK it was 6.0 mL/min/1.73 m^2^ in 1997, 7.5 in 2003 [27] and 6.9 in 2023 [28], and in the USA it increased between 2003 and 2010 and then decreased thereafter, being 9.5 mL/min/1.73 m^2^ in the 2020s [29, 30]. However, variability was observed between US states and within Europe, as estimated GFR (eGFR) was 8 mL/min/1.73 m^2^ in a multinational European contemporary cohort of elderly patients [31].

## LIMITATIONS

Some limitations of the GBD data should be acknowledged. Briefly, the GBD data are estimates, albeit using comprehensive data sources, including national death registries, nationally representative cohort studies and sophisticated modelling. Estimates are inevitably less precise in regions or disease areas where data sources are sparse [11]. The data sources are often based on a single eGFR sample without follow-up testing to confirm chronicity of CKD. There may be acquisition bias in reports based on those who have had blood or urine sampling performed, meaning the prevalence of CKD in people who have not been tested can only be estimated. In this round of GBD reporting on CKD, a deliberate choice not to focus on underlying cause of CKD was made on the basis that with better understanding of underlying aetiology of CKD, it is likely that previous reports overestimate the prevalence ‘hypertensive nephropathy’ as a cause of CKD [11, 32]. Additionally, the recent GBD report focused on adults, as data in paediatric populations are sparse and warrant dedicated study. Coding of cause of death will vary by country which may impact on the reported mortality due to CKD, although the code of death ensemble model [33] and burden of proof approaches used by GBD addresses these issues [34, 35]. Nevertheless, there remains debate around how many of the deaths attributable to CKD in the GBD reports are deaths ‘with’ CKD, rather than directly ‘attributable’ to CKD.

GBD should reconsider how it reports causes for CKD and KRT. The ERA Registry identified IKD/CAKUT as a cause of prevalent KRT in Europe more common than diabetes mellitus or hypertension (women) or as common as diabetes mellitus and more common than hypertension (men) [25]. Incorporating these highly prevalent genetic causes of KRT in future GBD iterations would enhance visibility, and support the recommendations by the Genes&Kidneys Working Group of the ERA to incorporate genetic testing when defining CKD with unexplained cause [CKDx; a phenomenon that is notably distinct from CKD of uncertain aetiology (CKDu)] [36]. Quantifying this major inherited cause may substantially reduce the proportion of cases unhelpfully classified as ‘other’ cause in Europe, which are as high as 46% to 48% in Central and Western Europe, with likely similar effects in other regions.

## CALL TO ACTION

The current burden and future growth of the burden of CKD and KRT calls for urgent action. Accounting for life expectancy estimates, people that will die from CKD by 2050 are now in their fifties and sixties. This age range is already used for population screening for colorectal cancer, a condition that will kill fewer people in Europe than CKD by the same date. One potential option for screening for CKD would be linking albuminuria screening for CKD to current cancer screening programs of occult blood in stool screening to facilitate the early detection of CKD in a potentially cost-effective manner [37, 38]. Albuminuria testing may also be expanded beyond people with traditional risk factors (diabetes, hypertension) to include people with other, under-recognized high risk conditions (e.g. cancer and HIV), and those apparently healthy individuals who are older or have GFR below values expected for age but above 60 mL/min/1.73 m^2^ [39–41]. Further consideration is required of how best to reframe ‘screening’ for CKD, and whether programmes continue to address albuminuria testing targeted at people at risk, or whether there is a case for wider population-based screening in people over a certain age. In this regard, interventions at the stage of pathologic albuminuria with preserved kidney function may delay the need for KRT by over two decades [4]. Moreover, in contrast to colorectal cancer screening, in which a positive test triggers a costly, invasive procedure, positive albuminuria tests may trigger a second, low-cost, non-invasive albuminuria test 3 months later that would already diagnose CKD if positive. Evidence to date suggests that screening for CKD is only cost effective in people at high risk (e.g. diabetes, certain ethnic groups). The economic case for screening is substantially strengthened by considering the additional benefit of identifying people with factors amenable to intervention to reduce risk of future cardiovascular events as well as progressive CKD [42, 43]. The recent upsurge in therapies available to nephrologists with sodium glucose cotransporter 2 inhibition, glucagon-like peptide-1 agonists and novel mineralocorticoid receptor antagonists, as well as more targeted therapies for glomerular disease suggest that CKD should be viewed as a treatable condition, rather than simply a cardiovascular risk factor and a precursor to KRT [44].

Overall, the kidney health community should advocate for compliance with the 2025 WHO kidney health resolution which is fully aligned with the contents of a recent KDIGO Controversies Conference on maintaining kidney health [45]. This resolution urges Member States, in accordance with their national context and priorities, to invest in health systems to integrate prevention, early detection and management of kidney disease into national health policies and inclusion of kidney management into universal health coverage benefit package, including the universal access to KRT [24].

## Data Availability

No new data were generated or analysed in support of this research.
